# Surgical management of anastomotic leakage related to ovarian cancer surgery: a narrative review

**DOI:** 10.3389/fsurg.2024.1434730

**Published:** 2024-09-11

**Authors:** Stefano Restaino, Sofia Schierano, Martina Arcieri, Barbara Costantini, Alice Poli, Sara Pregnolato, Giovanni Terrosu, Sergio Calandra, Marco Petrillo, Giulia Pellecchia, Alessandro Lucidi, Marko Klarić, Lorenza Driul, Vito Chiantera, Alfredo Ercoli, Cristina Taliento, Francesco Fanfani, Anna Fagotti, Giovanni Scambia, Giuseppe Vizzielli

**Affiliations:** ^1^ Department of Medical Area (DMED), Clinic of Obstetrics and Gynecology Unit, Santa Maria Della Misericordia Hospital, Azienda Sanitaria Friuli Centrale, Udine, Italy; ^2^ School in Biomedical Sciences, Gender Medicine, Child and Women Health, University of Sassari, Sassari, Italy; ^3^ Dipartimento per le Scienze Della Salute Della Donna, del Bambino e di Sanità Pubblic, Unit of Gynecologic Oncology, Fondazione Policlinico Universitario A. Gemelli IRCCS, Rome, Italy; ^4^ Liver-Kidney Transplant Unit, Department of Medical Area (DMED), Santa Maria Della Misericordia Hospital, Azienda Sanitaria Friuli Centrale, Udine, Italy; ^5^ Gynecologic and Obstetric Clinic, Department of Medicine, Surgery and Pharmacy, University of Sassari, Sassari, Italy; ^6^ Centre for Fetal Care and High-Risk Pregnancy, Department of Obstetrics and Gynecology, University of Chieti, Chieti, Italy; ^7^ Department of Obstetrics and Gynaecology, Clinical Hospital Center of Rijeka, Rijeka, Croatia; ^8^ Department of Health Promotion, Mother and Childcare, Internal Medicine and Medical Specialties (PROMISE), University of Palermo, Palermo, Italy; ^9^ Unit of Gynecology and Obstetrics, Department of Human Pathology of Adults and Childhood, University Hospital “G.Martino”, Messina, Italy; ^10^ Department of Obstetrics and Gynecology, University Hospital Ferrara, Ferrara, Italy; ^11^ Department of Obstetrics and Gynecology, University Hospitals Leuven, Leuven, Belgium

**Keywords:** ovarian cancer, debulking surgery, anastomosis leakage, stoma, rectosigmoid resection, risk factors

## Abstract

This narrative review describes the state of the art in the management of anastomotic leakage in ovarian cancer. Multiple surgical procedures, including bowel resection, are often required to achieve “optimal” cytoreduction in locally advanced ovarian cancer. Intestinal anastomosis is currently the most common way to restore bowel continuity. However, in some patients, a temporary protective stoma is indicated to prevent anastomotic leakage. This is an important issue to improve surgical outcomes and until recently there has been a lack of objective data to clarify the risk factors for anastomotic leakage. This review describes the risk factors for AL associated with surgery and compares the results of recent studies. We also review the current indications for placement of a protective ileostomy and treatment options for conservative management of AL. We present two examples of practical clinical AL risk calculators, in addition to the most assessed AL risk factor. To date, the decision-making processes that lead surgeons to perform a protective ileostomy are quite heterogeneous and based on the personal experience of the surgeon, mainly depending on individual training. Three different management options after colorectal anastomosis in OC are described: conservative management, diversion ileostomy and ghost ileostomy.

## 1 Introduction

Ovarian cancer (OC) is the most fatal of all cancers of the female reproductive organs. The mortality rate for OC is the highest of all gynecological malignancies, with a five-year survival rate of 43% (1). Multiple surgical procedures, including bowel resection, are often required to ensure the goal of no macroscopic residual disease or at least ≤1 cm of residual disease to achieve “optimal” cytoreduction (2, 3), regardless of patient age (4, 5). The literature reports that bowel resection is performed in 30%–70% of patients with advanced OC, with the rectosigmoid colon being the most commonly affected area, followed by the ileocecal stoma. Intestinal anastomosis is the traditional way to restore bowel continuity; however, a higher rate of postoperative complications cannot be excluded. A temporary protective stoma can reduce the severity of postoperative complications, but this procedure has disadvantages such as stoma-related complications and socio-psychological effects (6). The most significant complication associated with bowel resection is anastomotic leakage (AL). According to recent literature, it occurs at a median rate of 5.3% (7, 8). AL is a serious adverse event that could have a negative impact on patients’ overall prognosis and is associated with poorer perioperative outcomes, including longer hospital stay and delayed time to start adjuvant chemotherapy, which may contribute to negative survival outcomes (9). The ESGO Ovarian Cancer Guidelines do not recommend the routine use of protective stoma creation to reduce the risk of bowel complications in ovarian cancer patients undergoing bowel resection (10), as it is not without potential complications such as malnutrition, acute kidney injury, severe psychological effects and reduced quality of life (11). Prevention of anastomotic leakage is an important issue to improve surgical outcomes, and until recently there has been a lack of objective data to clarify the risk factors for anastomotic leakage. The primary aim of this review is to describe the risk factors for AL associated with surgery for locally advanced ovarian cancer. Secondly, the current indications for the placement of a protective ileostomy are reported. Finally, treatment options for conservative management of AL are described.

## 2 Methods

We conducted a narrative analysis of the published literature.

For narrative purposes, results are presented according to AL management:

- Risk factors for anastomotic leakage in advanced OC surgery;
- Decisions to treat with an ostomy as a prophylactic measure for AL;
- Conservative endoscopic management of AL.

### 2.1 Search strategy

A comprehensive search of the literature from January 2010 to July 2023 was conducted by using the following search strategy: “ovarian cancer” AND (“intestinal surgery” OR “bowel surgery” OR “ostom*” OR “leak*”) NOT (chemotherapy). Databases included pubmed, Ovid Cochrane Database of Systematic Reviews, and Scopus. Data were extracted into EndNote reference manager.

### 2.2 Eligibility criteria

All original articles written in English that reported quantitative and qualitative outcomes and study protocols were included, with no restriction on study design. Conceptual studies and any studies that did not mention bowel surgery as a complication of ovarian cancer surgery were excluded during the reading process.

### 2.3 Data extraction

Data were extracted by two investigators (S.S. and A.P.) independently for each eligible study. Disagreements were resolved by a third reviewer (S.R.) until consensus was reached. Full-text copies of these papers were obtained and the same reviewers independently extracted relevant data on study characteristics.

The initial search identified 272 studies. 104 records were excluded due to duplication, and 104 were excluded based on title and abstract screening. After applying the screening criteria, 72 articles were considered eligible for full-text reading, and after applying the exclusion criteria, 38 studies were selected for the final analysis.

Finally, thirteen studies (retrospective cohort study, prospective cohort study, systematic review and meta-analysis) are described in the results and summarised in the Supplementary Material.

All studies selected for this review met the following inclusion criteria:

- Comparison of outcomes of different therapeutic strategies;
- Available data on surgical complications;
- Medical data on patients;
- Only full-text articles were considered for inclusion.

The exclusion criteria for this review were as follows:

- Letters, editorials, case reports;
- Studies not published in English;
- Studies with missing outcome data.

## 3 Results

### 3.1 Risk factors for anastomotic leakage in advanced OC surgery

**Figure 1:**
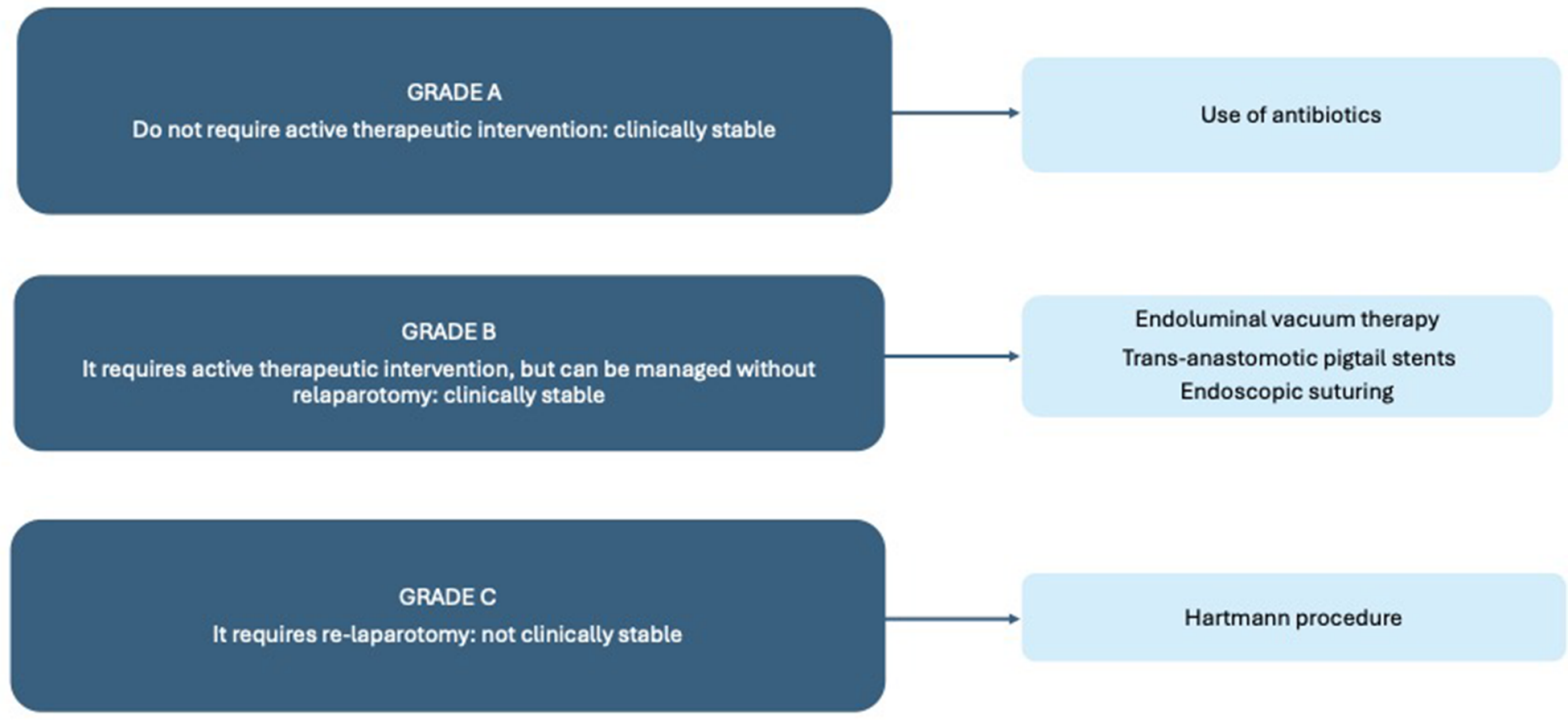
The risk factors for AL after multivariate analysis in the studies included in this review.

Risk factors for AL have recently been investigated by several studies, and according to a retrospective analysis of a multicenter cohort by V. Lago et al. (12) the independent risk factors for AL (Figure 1) in multivariate analysis are:

- Age at surgery: a 1-year increase in age was associated with a 1.046-fold increase in the odds of anastomotic leakage.
- Preoperative albumin level <30 mg/dl: as the serum albumin level increases (better nutritional status), the risk of AL decreases (*p* = 0.027); a one-unit increase in serum albumin level is associated with a 0.62 decrease in the odds of anastomotic leakage.
- Multiple small bowel resection: small bowel resection is associated with a 3.54 increase in the odds of anastomotic leakage compared to no bowel resection in addition to large bowel resection (*p* = 0.019).
- Manual anastomosis: associated with 8.36-fold increased odds of anastomotic leakage compared with the use of both anastomotic techniques. According to these results, stapled anastomosis seems to be justified as an elective technique to prevent AL.
- Distance of the anastomosis from the anal verge: as the distance increases, the risk of AL decreases (*p* = 0.018).

Lago V, Fotopoulou C et al. also offer a free access app: The Anastomotic Leak Prognostic Score “The OVA-LEAK Score”, which provides a risk percentage calculator based on multivariate logistic regression analysis of preoperative and intraoperative risk factors. The OVA-LEAK Score application asks for the following variables: age, BMI, insulin-dependent diabetes, neoadjuvant chemotherapy, albumin serum level, additional bowel resection, type of anastomosis (stapled, hand sutured), use of stoma (ghost or diverted ileostomy), distance from colorectal anastomosis to anal verge, intraoperative red blood transfusion, operation time and use of hyperthermic intraperitoneal chemoperfusion (HIPEC) (12).

The first systematic review and meta-analysis of preoperative and intraoperative risk factors for anastomotic leakage (AL) after OC bowel resection and anastomosis has been presented the literature by Valenti G, Vitagliano A et al. (7); it found that:

- Preoperative albumin level <30 mg/dl is a risk factor for AL (*p* = 0.009).
- Multiple bowel resections are a risk factor for AL (*P* = 0.03), a subanalysis of the type of bowel resection was not applicable.
- Primary cytoreduction is a risk factor for AL (*p* = 0.03)
- ASA score is not a risk factor for AL (*P* = 0.47)
- Suboptimal debulking is not a risk factor for AL (*P* = 0.30)
- Ascites is not a risk factor for AL (*P* = 0.44)
- Protective ileostomy is not a risk factor for AL (*P* = 0.68)

**Figure 2:**
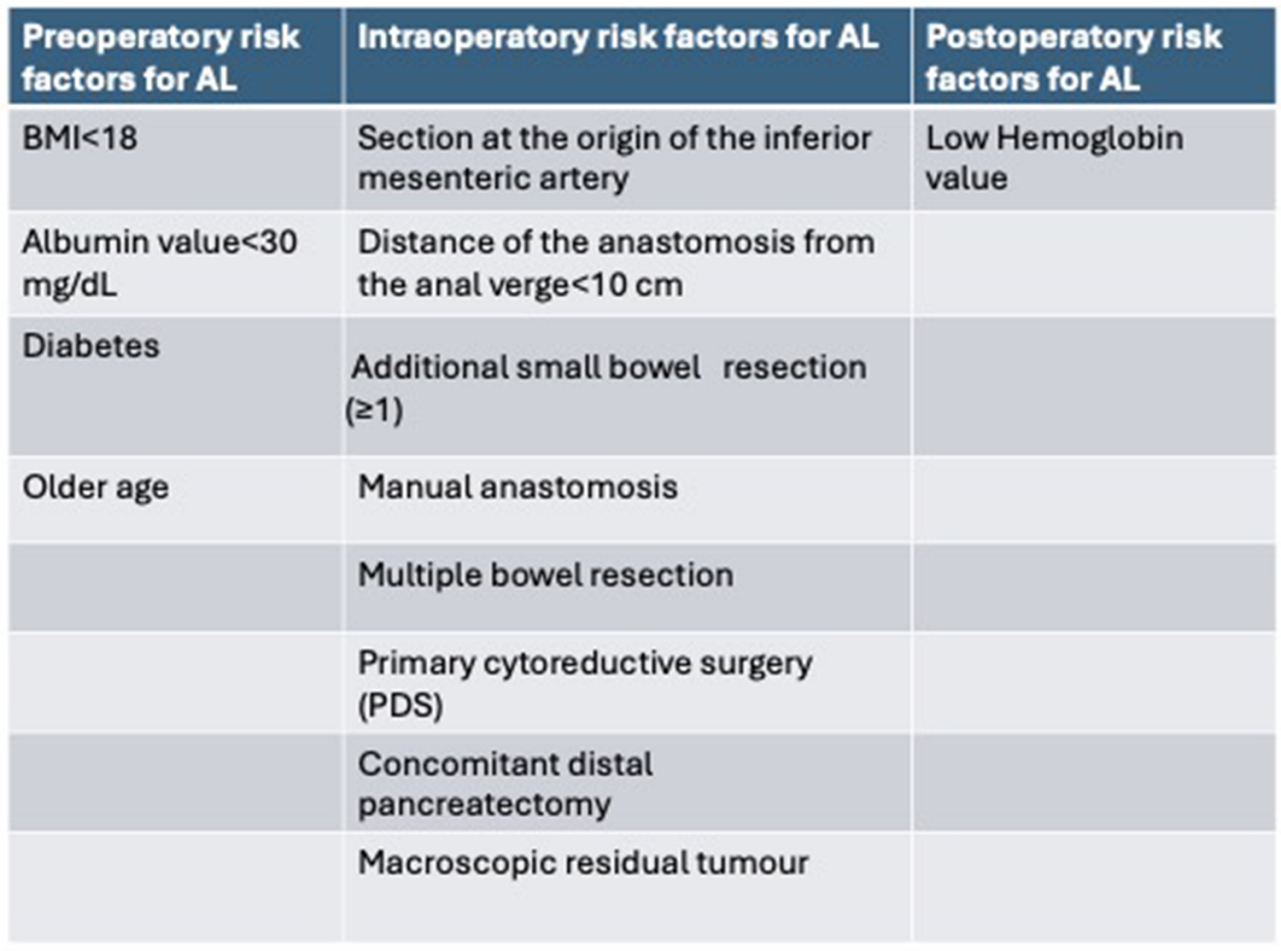
The grading of AL and its management.

In conclusion, only preoperative serum albumin level <30 mg/dl, multiple bowel resections and primary cytoreduction have been identified as risk factors that may independently increase the rate of AL (Figure 2) (7).

According to Costantini B, Vargiu V et al. (13), intraoperative independent risk factors for AL were observed to be a distance to the anastomosis from the anal verge of <10 cm (*p* = 0.004) and the section of the inferior mesenteric artery at its origin (*p* = 0.008). Independent preoperative risk factors for AL were a preoperative albumin level of <30 mg/dl and a body mass index (BMI) of <18 kg/m^2^ (13).

The study by Kim JH, Han WH et al. (9) was conducted in the largest Asian population-based cohort to their knowledge and identified the following three new independent variables as risk factors for AL: diabetes, concomitant distal pancreatectomy and postoperative residual disease. They also suggested the use of a nomogram in clinical practice based on multivariate analysis: diabetes (OR 3.32; 95% CI, 1.21–9.09; *p* = 0.02), distal pancreatectomy (OR 6.32, 95% CI 2.22–18.04; *p* = 0.001), residual disease (OR 7.12, 95% CI 3.24–15.66; *p* < 0.001) and anastomotic distance from the anal verge <10 cm (9).

The study by Sánchez-Iglesias JL, Gómez-Hidalgo NR et al. (14) investigated the impact of discontinuing mechanical bowel preparation in advanced OC surgery as part of the ERAS programme. The study was designed to compare patients undergoing cytoreductive surgery with bowel resection before and after the era of the ERAS protocol, with particular attention to the incidence of anastomotic leakage, abscess and fistula. A comparison of postoperative bowel complication outcomes between the mechanical bowel preparation (MBP) and no bowel preparation (NMBP) groups shows a non-significant difference, with a 12% rate of anastomotic-related complications in each group. Specifically colorectal anastomotic leakage occurred in 4 patients (5.3%) in the NMBP group compared to one patient (2.6%) in the MBP group (*p* = 0.11; Fisher exact test), specifically:

• Small bowel leak was observed in two patients (2.7%) in the NMBP group and none in the MBP group.
• Abscess occurred in 4 patients with MBP (10.3%) and in one patient with NMBP (1.3%).
• None of the patients with MBP had a bowel fistula, but two patients with NMBP did (2.8%).

The overall reintervention rate was 17 out of 114 (14.9%), with 10.3% in the MBP group and 17.3% in the NMBP group (*p* = 0.314, chi-squared test), with no significance in the range of days to reintervention (*p* = 0.461, Mann–Whitney *U*). The main cause of reintervention was AL, which occurred in a total of 7 patients (6.1%), six in the NMBP group (8.0%) vs. one in the MBP group (2.6%; *p* value = 0.522, Fisher exact test) (14).

T. Bartl, R. Schwameis et al. (15) analyzed a cohort of 350 patients, of whom 192 patients (54.9%) underwent at least one bowel resection. Risk factors for AL were calculated using logistic regression models; the AL rate was 4.7% in patients with advanced EOC who underwent cytoreductive surgery, including patients with multiple colectomies. The AL rate was 1.9% in patients with isolated rectosigmoid resection. In univariate analysis, the number of anastomoses per operation was associated with the occurrence of AL (*P* = 0.04). In multivariable analysis, rectosigmoid resection with additional colon resection was associated with a higher risk of AL compared with isolated rectosigmoid resection [*P* = 0.046; OR 7.23 (CI, 1.04–50.39)] (15).

The study by Son JH, Kim J et al. (16) compared two surgical techniques and postoperative outcomes for rectosigmoid colectomy with cytoreduction for advanced OC. The aim of the study was to evaluate the clinical outcomes of close rectal dissection (CRD) compared with total mesorectal excision (TME) as a procedure for dissection of the posterior rectum. Total mesorectal excision (TME) has traditionally been used for rectosigmoid resection. It is widely accepted as the preferred technique for the surgical removal of rectal cancer. Because the procedure completely removes the mesorectal tissue, TME results in lower rates of local recurrence of bowel cancer. However, there are few studies on techniques for posterior dissection of the rectosigmoid colon in patients with ovarian cancer. Perioperative outcomes were evaluated in patients who underwent TME or CRD. Risk factors for anastomotic leakage in univariate analysis were postoperative hemoglobin level, subtotal colectomy and total mesorectal excision vs. close rectal dissection. In multivariate analysis, only postoperative hemoglobin level remained as an independent risk factor. The pelvic recurrence rate was not different between the two groups (*p* = 0.663). Progression-free survival was not significantly different between the two groups (median, 22 vs. 26 months; *p* = 0.790) (16).

### 3.2 Decisions to treat with an ostomy as a prophylactic measure for AL

The study by Gockley AA, Fiascone S et al. (3) describes the outcomes of patients undergoing cytoreductive surgery for advanced OC at two large academic centers. The rate of AL in this study was 3.6%, considered a good indicator of a quality center as it is comparable to previous reports of 1%–8%. A subset of factors was included as independent variables in a multivariable logistic regression model to assess independent intraoperative factors that may influence the decision to create an ostomy at the time of cytoreductive surgery. These factors were: age, stage, Aletti Surgical Complexity Score (17), comorbidity index, preoperative CA125 and preoperative albumin. Multivariate analysis showed that only a high Aletti score based on surgical procedures such as omentectomy (1 point), pelvic lymphadenectomy (1 point), paraortic lymphadenectomy (1 point), pelvic peritoneal stripping (1 point), abdominal peritoneal stripping (1 point), rectosigmoidectomy and colo-anal end-to-end anastomosis (3 points), colon resection (2 points), diaphragmatic stripping resection (2 points), splenectomy (2 points), liver resection (2 points) and small bowel resection (1 point) were associated with an increased risk of ostomy formation. As a result, patients with an ostomy had a median progression-free survival of 11.6 months, which was not significantly different from patients without an ostomy who had a progression-free survival of 16 months (*p* = 0.37), and there were no significant differences in length of hospital stay or delay in postoperative chemotherapy between patients with and without an ostomy. It was highlighted that ostomy creation did not affect the optimal resection rate between the ostomy and no ostomy groups (84% vs. 92.1%, *p* = 0.39) (3).

In the most recent review by He J, Li J et al. (6), they selected studies that evaluated ostomy in OC and analyzed the reasons why surgeons decided to perform a protective ostomy. They reported on a total of 2,868 patients included in 12 articles, with ostomy rates ranging from 1.7% to 58%. The most common reasons for the surgeon to choose a protective ostomy were longer operative time and lower positioned anastomosis, previous treatment with bevacizumab, additional bowel resection, intraoperative red blood transfusion, high Aletti score, poor tissue quality, non-tension-free anastomosis at the staple line, spillage on air test, preoperative colonic obstruction, small bowel anastomosis, poor bowel preparation, ascites >500 ml, previous pelvic radiation (6).

Regarding the option of ghost ileostomy to manage bowel resection in patients with OC, to our knowledge the first study in the literature is by Lago V, Flor B et al. (18): “Ghost Ileostomy (GI) in Advanced Ovarian Cancer: A Reliable Option”, describing the experience of their center. The so-called virtual or ghost ileostomy is a pre-stage ostomy that can be easily exteriorized if an anastomotic leak is suspected. This avoids the serious consequences of anastomotic leakage. On the other hand, a real ileostomy can be avoided in patients who do not develop anastomotic leakage. They confirm that ghost ileostomy is a real option that can reduce the number of ileostomies performed in ovarian cancer without increasing morbidity. Rectoscopy was performed on postoperative day 5 ± 1 to check the integrity of the anastomosis. In 2 cases the rectoscopy showed a leak without clinical symptoms. No other ghost ileostomy (GI) or diverting ileostomy (DI) related complications were observed during the postoperative course. This study concludes that ghost ileostomy appears to be a safe, feasible and reproducible technique with no significant impact on operative costs (18).

Lago V, Sanchez-Migallón A et al. (19) compared three different management options after colorectal anastomosis in OC: conservative management, diversion ileostomy and ghost ileostomy. A total of 133 patients were included in the analysis. The rate of anastomotic leakage was 5.6% after watchful waiting (4/72), 5.3% after diversion ileostomy (1/19) and 4.8% after ghost ileostomy (2/42), with no differences in the rate of AL (*p* = 0.98). All patients with AL in the conservative group required intensive care unit (ICU) admission and laparotomy for ostomy creation (75% end colostomy). In contrast, neither the diverted ileostomy nor the ghost ileostomy group required ICU admission, and the ghost ileostomy group did not require laparotomy for ostomy creation. Anastomotic leakage led to the death of two patients in the conservative group (50%), whereas none of the patients in the diverted ileostomy or ghost ileostomy groups died (19).

The retrospective study of 515 patients by Costantini B, Vargiu V et al. (13) made a comparison of risk factors for stoma diversion and risk factors for anastomotic leakage, so they analyzed factors associated with the surgeon's decision to perform an ostomy and factors retrospectively associated with AL in their study cohort. It was found that of the factors associated with stoma diversion (more than two bowel resections, distance of the anastomosis from the anal verge <10 cm, operation time >300 min and intraoperative transfusions), only more than two bowel resections and distance of the anastomosis from the anal verge <10 cm was significantly associated with the risk of AL. It was also highlighted that the creation of a protective stoma does not prevent intestinal AL, but rather influences the severity of the complication and therefore the type of surgical treatment required to resolve the leak. In detail, the postoperative surgical report of AL cases described seven patients (46.7%) with a diversion stoma and eight (53.3%) without a diversion stoma. None of the patients without a diversion stoma and AL were managed conservatively and all of these patients required re-intervention: six out of eight (75%) were treated with the Hartmann procedure and the other two received an ileostomy. In contrast, the seven patients with a diverted ostomy and AL, of whom 3 (42.9%) required conservative management with drainage plus broad-spectrum antibiotics, the remaining patients 4 (57.1%) underwent re-operation with resection of the anastomotic complex and a Hartmann colostomy. In fact, there was a higher percentage of Hartmann colostomies in the group of patients without a protective stoma than in those with a protective stoma (75% vs. 57%), and therefore a greater likelihood of failure to recanalize the bowel (13).

To guide the decision to place a protective ileostomy after rectosigmoid resection, Kalogera E, Nitschmann CC et al. (11) developed a prospectively tested algorithm. A cohort was selected based on at least one of the following eight criteria for prophylactic bowel diversion in patients undergoing rectosigmoid resection at the time of OC cytoreduction. These criteria were: preoperative albumin ≤30 mg/dl, prior pelvic irradiation, rectosigmoid resection, and additional colon resection, anastomosis ≤6.0 cm from the anal verge, evidence of bowel compromise, and intraoperative leakage on air leak test or gross contamination. In this prospectively selected cohort of 77 patients studied, 27 patients (35.1%) received a diversion ostomy and showed a statistically significant reduction in the rate of AL (1.3%) compared to a historical control rate of 7.8% when applying the study criteria for diversion described above (11).

In 2020 Lago V, Fotopoulou C et al., one year after the publication of “Risk factors for anastomotic leakage after colorectal resection in ovarian cancer surgery: A multicenter study” (12), they published “Indications and practice of diverting ileostomy after colorectal resection and anastomosis in ovarian cancer cytoreduction” (20), which retrospectively analyzed the same multicenter cohort of patients. The rate of AL was 13.9% (15/108) vs. 8.9% (31/349) in the ileostomy group vs. no ileostomy group (*p* = 0.184). They analyzed the factors associated with the performance of ileostomy diversion. On multivariate analysis, the following factors were independently associated with the use of a DI: previous treatment with bevacizumab (*p* = 0.01), additional bowel resection (*p* = 0.001), prolonged operation time (*p* = 0.001) and intraoperative red blood transfusion (*p* = 0.001). These factors appear to influence the surgeon's view and condition the occurrence of DI, whereas it has previously been shown that none of these factors are actually significantly associated with AL, with the sole exception of multiple bowel resections (20). Assuming a 7% AL rate cut-off as acceptable, the risk factor-based prediction model was applied to patients undergoing DI after colectomy and anastomosis. Up to 51.8% of them were calculated to have an AL risk <7%, and therefore DI may not have been necessary based on a well-defined risk factor policy (20).

These results provided a hypothesis that was validated in 2022 by a retrospective multicenter cohort follow-up study conducted by the OVA-LEAK Collaborative Group of Lago V, Segarra-Vidal B et al. (21) including patients from 12 cancer centers, to validate the OVA-LEAK prediction score for AL with the aim of avoiding unnecessary DI formation as much as possible. According to the OVA-LEAK calculator, a cut-off of 22.1% was chosen to consider a patient at risk of having a leak and being selected for diversion. Applying the OVA-LEAK calculator to the cohort, up to 22.5% of patients would have undergone a diversion ileostomy and 47% (18/40) of ALs would have been “protected” by the stoma. Conversely, if only the “clinical criteria and surgeon's decision” to do or not to do a diversion ileostomy is considered, only 12.5% (5/40) of leaks would be “protected” by a stoma, with a diversion ileostomy rate of up to 24.3% (21).

According to the systematic review and meta-analysis by Santana BN, Garcia Torralba E et al. (22) there was no difference in the rate of AL, urgent reinterventions and mortality due to AL in ostomy patients compared with non-ostomy patients. All 17 studies were included, with a total of 2,719 patients. There were 475 patients with an ostomy and 2,244 patients without an ostomy. The AL rate was 6.5% (*n* = 31) in the ostomy group and 8.5% (*n* = 190) in the non-ostomy group. The pooled OR for the studies was 1.01 (95% CI = 0.60–1.70; *p* = 0.980), indicating that ostomy formation was not significantly associated with less AL compared with non-ostomy patients (22).

### 3.3 Conservative endoscopic management of AL

Not all AL requires immediate intervention, and a robust classification system and evidence-based management algorithms are now in use to help gynecological oncologists support and manage OC patients with colorectal complications. To summarize the algorithm, there are three different grades of AL: Grade A does not require active therapeutic intervention and is managed with antibiotics and a watch-and-see approach, Grade B requires active therapeutic intervention but can be managed without relaparotomy, and Grade C requires relaparotomy (23).

Surgical management is required if the patient has peritonitis or severe sepsis and is not clinically stable, with relaparotomy based on the Hartmann procedure as the gold standard, with the risk of subsequent leakage and the main disadvantage of a higher risk of permanent stoma (24).

Other approaches may be considered instead of relaparotomy in less clinically compromised patients with grade B AL, in clinically stable patients, and in low-grade septic abscesses (<3 cm). Endoluminal vacuum therapy, trans-anastomotic pigtail stents or endoscopic suturing are currently used in clinical practice. There is a paucity of literature comparing these approaches (25), however, a retrospective study by Thiruvengadam et al, patients who received combined therapy (endoscopic drainage with local closure) had the best clinical success rates (26).

The placement of self-expanding stents to plug the repair of wall integrity has an estimated success rate of around 70%, but there remains the potential problem of stent migration, which therefore needs to be monitored over time. However, endoscopic stenting in the early postoperative management of anastomotic complications after colorectal surgery should be considered as a safe and often effective alternative to surgery. The last of these possible endoscopic techniques is the placement of a sterile sponge that works with a suction system at negative pressure over time to dry the abscess; however, this technique requires several endoscopic sessions (11 ± 6), sponge change every two days, and a long hospital stay (30 ± 12) (27).

## 4 Discussion

Our review aimed to summarize the risk factors for AL associated with surgery for advanced ovarian cancer, the most recent indications for the placement of a protective ileostomy and the conservative management options for AL management.

A multidisciplinary approach to the management of AL is required, and predictive models may be helpful in the clinical setting. This review presents two examples of practical clinical risk calculators for AL. The study by Kim JH, Han WH et al. (9) presented a free web-based nomogram that could guide preoperative patient counselling and intraoperative decision making. The calculated basal risk was 9.12% and increased with the addition of variables such as diabetes, distal pancreatectomy, anastomotic level from the anorectal margin <10 cm and macroscopic residual tumor. These findings may be explained as follows:

- Diabetes is one of the most robust factors influencing the risk of AL, but due to the heterogeneity of the cohort in the assessment of AL risk, diabetes remains a confounding variable that requires careful interpretation
- In their cohort, distal pancreatectomy was newly identified as a risk factor for AL in multivariate analysis and was performed in 3.3% (26/770) of patients, but there is a lack of data on the incidence of distal pancreatectomy in other studies. Approximately 30% of patients who have undergone distal pancreatectomy develop a pancreatic leak, and the spillage of amylase into the abdomen can lead to serious abdominal infections.
- Macroscopic residual disease as a risk factor for AL may be associated with high surgical complexity.

According to the authors the nomogram can be used in clinical practice as a tool to estimate the risk of anastomotic leakage after rectosigmoid resection for ovarian cancer. However, further evidence is needed for these identified risk factors (9).

The technique of close rectal dissection during posterior rectal dissection preserves the rectal vessels in the mesorectum, it could be a reasonable alternative in terms of rectal perfusion without compromising oncological outcome (15).

The OVA-LEAK Collaborative Group in 2022 validated the anastomotic leak prognostic score “The OVA-LEAK score” in an external cohort and confirmed that their predictive algorithm is more sensitive than subjective surgeon clinical criteria and does not increase the rate of ileostomy diversion. This is the first example of risk stratification algorithms that clearly guide the creation of a DI in AOC (21).

Although several statistical models have been proposed to estimate the risk of AL after colectomy, it is difficult to predict which patients will develop AL. Artificial intelligence (AI) algorithms have been used to develop a powerful model for predicting AL in a cohort of patients with rectal cancer, with promising results. However, to date no clinical trials have investigated the use of AI in OC surgery. The use of AI in clinical practice will help surgeons to identify patients at low risk of AL during colectomy and avoid unnecessary temporary ileostomies. The authors developed a web application to assess the risk of AL in real time during the intraoperative period, taking into account several factors; such as: age, BMI, comorbidities, previous lower abdominal surgery, tumor obstruction, pre-operative hemoglobin level (g/dl), preoperative albumin level (g/L), electrolyte disorder, tumor size (cm), distance between the lower edge of the tumor and the anal edge (cm) and operative time (min) (28).

The Enhanced Recovery After Surgery (ERAS) program for advanced OC surgery has recently been introduced into clinical practice. This represents an important change in the management of advanced OC, with the discontinuation of routine mechanical bowel preparation due to its distressing side effects for patients. The ERAS Society Guidelines for Perioperative Care in Gynecological Oncology Surgery attempt to standardize care by providing a reproducible approach for patients undergoing gynecological surgery. Most of the data supporting ERAS comes from colorectal surgery, and it remains unknown whether ERAS is also safe for ovarian cancer patients undergoing rectosigmoid resection with anastomosis as part of cytoreductive surgery (29). Considering the current evidence, the introduction of protocols for preoperative nutritional support for patients is strongly supported and it is suggested that surgery should be carefully tailored to maximize blood supply to the anastomoses (30).

The retrospective study by Sánchez-Iglesias JL, Gómez-Hidalgo NR et al. did not show a significantly higher risk of mortality or infection in the NMBP group compared with the MBP group, suggesting that preoperative MBP may not be essential in OC surgery, especially in OC surgery with bowel resection. This is the first trial to assess the benefit of MBP in surgery for advanced ovarian cancer. It would be important to understand whether NMBP is a specific risk factor for AL and relaparotomy (14).

The use of novel technologies such as near-infrared imaging to assess anastomotic perfusion after colorectal anastomosis is encouraged, as it can provide real-time insight into whether bowel diversion should be performed. The systematic review by Spagnolo E, Zapardiel I et al. evaluating the role of indocyanine green (ICG) fluorescence imaging for intraoperative bowel assessment in gynecological surgery identified only one study involving 82 operations for advanced ovarian cancer. Intraoperative ICG fluorescence imaging has been shown to be an effective tool in colorectal surgery to assess blood supply during bowel anastomosis to reduce anastomotic leakage (AL) rates in primary and secondary cytoreduction of gynecological malignancies (31).

According to Costantini B, Vargiu V et al. (13), what drives a surgeon to perform a diversion ostomy is the evidence-based prevention of high-grade AL requiring demolition surgery and relaparotomy. There are conflicting reports in the literature regarding short-term mortality and ostomy performance; although several studies have identified AL as a negative prognostic factor for 90-day mortality and overall survival, a single-center, retrospective, observational cohort study published in 2022 reported data showing that AL had no effect on overall patient survival (OS). The difference between the AL and no-AL groups was not statistically significant (HR 1.767, 95% CI 0.869–3.594, *p* = 0.116), with median OS of 28 months and 50 months, respectively (13).

Ostomy formation has traditionally been associated with a delay in the starting of chemotherapy, which is associated with a worse prognosis and overall survival. However, the study by Gockley AA, Fiascone S et al. (3) described the outcomes of patients undergoing colorectal surgery with ostomy formation. It was found that there was no difference in the initiation of postoperative chemotherapy and median postoperative length of stay between the two cohorts of patients.

According to “Protective ostomies in ovarian cancer surgery: a systematic review and meta-analysis” by Navarro Santana B, Garcia Torralba E et al., the AL rate was not significantly associated with ostomy creation; however, this result may be explained by the fact that many trials had to correctly compare the two cohorts of patients (ostomy vs. non-ostomy), which led to a lower quality of the included trials. Clinical heterogeneity between trials was high due to the inclusion of different types of bowel resection, types of ostomy, types of surgery, types of cytoreductive surgery and the definition of AL. In addition, no prospective trials were included. Therefore, the benefit of a protective ostomy remains an open question, and these data are useful to reiterate the importance of standardizing the use of ostomy creation in ovarian cancer surgery (22).

Ghost ileostomy has already been proposed as an alternative to diversion ileostomy in the treatment of colorectal cancer because of its advantages over DI, and recently this procedure has been experimented with in gynecological surgery. The use of the ghost ileostomy in combination with close postoperative monitoring allows early and subclinical diagnosis of leakage (32).

This present narrative review describes the state of the art in the management of anastomotic leakage in ovarian cancer. The findings reported highlight that, to date, the factors associated with ostomy formation and the risk factors for AL are very different. Furthermore, the decision-making processes that lead surgeons to perform a protective ostomy are quite heterogeneous and based on the surgeon's personal experience, mainly depending on individual training, tradition and perception of the risk-benefit balance. As future directions, this study highlights the need to conduct larger prospective studies to adapt models of AI for AL risk in the surgical management of OC and to validate risk scores assessing the independent risk factors for AL in OC.
